# Photoprotective Effect of Ultrasonic-Assisted Ethanol Extract from *Sargassum horneri* on UVB-Exposed HaCaT Keratinocytes

**DOI:** 10.3390/antiox13111342

**Published:** 2024-11-01

**Authors:** Kirinde Gedara Isuru Sandanuwan Kirindage, Arachchige Maheshika Kumari Jayasinghe, Chang-Ik Ko, Yong-Seok Ahn, Soo-Jin Heo, Eun-A Kim, Nam-Ki Cho, Ginnae Ahn

**Affiliations:** 1 Department of Food Technology and Nutrition, Chonnam National University, Yeosu 59626, Republic of Korea; 2 Choung Ryong Fisheries Co., Ltd., Jeju-si 63612, Republic of Korea; rhckddlr01@naver.com (C.-I.K.); ecoil@hanmail.net (Y.-S.A.); 3 Jeju International Marine Science Center for Research & Education, Korea Institute of Ocean Science & Technology (KIOST), Jeju-si 63349, Republic of Korea; sjheo@kiost.ac.kr (S.-J.H.); euna0718@kiost.ac.kr (E.-A.K.); 4 College of Pharmacy, Chonnam National University, Gwangju 61186, Republic of Korea; cnamki@jnu.ac.kr; 5 Department of Marine Bio-Food Sciences, Chonnam National University, Yeosu 59626, Republic of Korea

**Keywords:** *Sargassum horneri*, photoprotective effect, apoptosis, oxidative stress, antioxidant capacity, ultrasonic extraction

## Abstract

The present study investigated the photoprotective effect of the ultrasonic-assisted ethanol extract (USHE) from *Sargassum horneri*, a brown seaweed containing fucosterol (6.22 ± 0.06 mg/g), sulfoquinovosyl glycerolipids (C_23_H_43_O_11_S, C_25_H_45_O_11_S, C_25_H_47_O_11_S, C_27_H_49_O_11_S), and polyphenols, against oxidative damage in ultraviolet B (UVB)-exposed HaCaT keratinocytes. USHE indicated antioxidant activity in ferric-reducing antioxidant power (FRAP) and 2,2-diphenyl-1-picrylhydrazyl (DPPH) radical scavenging. After screening experiments, 15.6, 31.3, and 62.5 µg/mL concentrations of USHE and ascorbic acid as positive control were selected to be used throughout the investigation. USHE increased cell viability by markedly reducing the production of intracellular reactive oxygen species (ROS) in UVB-exposed HaCaT keratinocytes. Additionally, USHE reduced the apoptosis and sub-G_1_ cell population and increased the mitochondrial membrane potential. Moreover, USHE modulated the protein expression levels of anti-apoptotic molecules (Bcl-xL, Bcl-2, and PARP) and pro-apoptotic molecules (Bax, cleaved caspase-3, p53, cleaved PARP, and cytochrome C). This modulation accorded with the upregulation of cytosolic heme oxygenase (HO)-1, NAD(P)H quinone oxidoreductase 1 (NQO 1), and nuclear factor erythroid-2-related factor 2 (Nrf2), collectively known as components of the antioxidant system. These findings suggest that USHE has a photoprotective effect on UVB-exposed HaCaT keratinocytes and can be utilized to develop cosmeceuticals for UVB protection.

## 1. Introduction

The increasing destruction of the ozone layer allows ultraviolet (UV) radiation (wavelengths ranging from 290 to 400 nm) to penetrate the Earth’s surface [1,2]. Among these, ultraviolet B (UVB) radiation, with wavelengths ranging from 290 to 320 nm [3], serves as a crucial catalyst for vitamin D synthesis in the skin [4]. Although this UVB exposure facilitates beneficial processes in the body, long-term exposure leads to reactive oxygen species (ROS) production within cells in the epidermis and dermis [1,5]. ROS, including superoxide radicals and hydrogen peroxide, function as signaling molecules and contribute to various cellular processes by altering the activity of metabolic enzymes and transcription factors [6]. ROS work as crucial secondary messengers that regulate various signaling pathways involved in inflammation, cell proliferation, and apoptosis [7]. For instance, ROS are involved in the activation of transcription factors like nuclear factor kappa-light-chain-enhancer of activated B cells (NF-κB) and nuclear factor erythroid 2-related factor 2 (Nrf2) signaling [8]. These factors are crucial for the cellular response to oxidative stress and aid in the regulation of genes that protect against oxidative damage [8]. Moreover, well-known ROS/H_2_O_2_ is recognized as modulating numerous signal transduction events that directly influence cellular responses to stress and metabolic demands [9].

However, the excessive production of ROS due to prolonged or intense exposure to UVB rays can overwhelm the natural defense mechanisms of antioxidants in skin cells. This stress significantly impacts cellular elements, including lipids, proteins, and DNA, and ultimately induces apoptosis, known as programmed cell death [6,10]. Disrupted DNA integrity contributes to a range of degenerative skin disorders, including sunburn, suntan, blisters, erythema, and melanomas [6]. Given that UVB exposure is unavoidable, it is essential to comprehend the delicate balance between potential benefits and risks to maintain better (skin) health. This understanding highlights the need for comprehensive sun protection strategies that include using antioxidant-rich skincare products, protective clothing, and careful sunscreen application to reduce the harmful effects of UVB radiation [11]. Recent investigations have increasingly focused on refining protective materials derived from safe, less toxic, and readily accessible bioresources.

As part of this evolving research field, revealing the molecular mechanisms underlying natural products’ biological functions is essential to exploring potential solutions [11]. Notably, studies have identified marine resources as rich sources consisting of various bioactive components including polysaccharides, phenols, sterols, proteins, and a variety of pigments exhibiting photoprotective effects [12].

Sargassum, a genus of brown macroalgae within the order Fucales of the Phaeophyceae class, has attracted significant attention for its bio-functional components, such as meroterpenoids, phlorotannins, sulfated polysaccharides, sterols, and glycolipids [13,14]. In particular, *Sargassum horneri*, an edible brown alga that is highly abundant along the coasts of Jeju Island in South Korea, has gained attention due to its high content of bioactive compounds. Reports have demonstrated that *S. horneri* has drawn more interest in studies of anti-inflammatory, antioxidant, anti-allergy, and anticancer agents [14,15].

We critically evaluated the anti-inflammatory effect of fucosterol isolated from *S. horneri* on human dermal fibroblasts [14], the anti-allergic effect of fucosterol isolated from *S. horneri* on bone marrow-derived cultured mast cells, and passive cutaneous anaphylaxis in mice in previous studies [16]. This study was designed to maximize target bioactive compound(s)/fucosterol while reducing the extraction time by employing an ultrasonic-assisted extraction technique. The ultrasonic-assisted ethanol extraction method employs high-frequency sound waves to improve solvent penetration into the plant matrix, which accelerates and enhances the extraction process. As reported, this technique not only shortens extraction times and requires lower temperatures but also better preserves thermally sensitive compounds compared to traditional extraction methods [17]. Moreover, this investigation aims to elucidate the photoprotective effects of the ultrasonic-assisted ethanol extract (USHE) from *S. horneri* on UVB-exposed HaCaT keratinocytes. It is hypothesized that USHE modulates intracellular ROS production, facilitating recovery from mitochondria-mediated apoptosis and downregulating oxidative stress via the Nrf2/heme oxygenase (HO)-1 signaling pathway in UVB-exposed HaCaT keratinocytes.

## 2. Materials and Methods

### 2.1. Materials

Dulbecco’s modified eagle medium (DMEM), penicillin/streptomycin mixture, and fetal bovine serum (FBS) were purchased from GibcoBRL (Grand Island, NY, USA). Folin and Ciocalteu’s phenol reagent, 2,2-diphenyl-1-picrylhydrazyl (DPPH), 3-(4,5-dimethylthiazol-2-yl)-2,5-diphenyltetrazolium bromide (MTT), 2′,7′-dichlorodihydrofluorescein diacetate (DCF-DA), dimethyl sulfoxide (DMSO), bovine serum albumin (BSA), propidium iodide (PI), acridine orange (AO), ethidium bromide (EB), ascorbic acid (AA), agarose, and gallic acid were bought from Sigma-Aldrich (St. Louis, MO, USA). D-glucose was purchased from Junsei Chemical Co., Ltd. (Tokyo, Japan). Skim milk powder was obtained from BD Difco^TM^ (Sparks, MD, USA). A BCA protein assay kit, NE-PER^®^ nuclear and cytoplasmic extraction kit, 1-Step transfer buffer, Pierce™ RIPA buffer, and a protein ladder were purchased from Thermo Fisher Scientific (Rockford, IL, USA). Primary and secondary antibodies needed for the Western blot analysis were bought from Cell Signaling Technology (Beverly, MA, USA). The remaining chemicals and reagents used in the study were analytical grade.

### 2.2. Ultrasonic-Assisted Ethanol Extraction

*S. horneri* was obtained from Jeju Island, South Korea. The sample was identified by the Biodiversity Research Institute in Jeju, South Korea. The sample was cleaned with running water at first. Afterward, samples were dried in the ventilated room at room temperature (27.5 ± 2.0 °C) and stored at room temperature for three weeks until extraction began. After soaking the 200 g of dried sample for 1 h with distilled water, 30 L of 50% ethanol was poured into the ultrasonic apparatus (MD-1200PG, Mirae ultrasonic, KR) along with the sample. The ultrasonic strength used in this extraction was 28 kHz with output power at 1200 W, the stirring speed was 60 rpm, and the initial temperature of the extract was 4 °C. The average temperature during the extraction process was maintained within 4 ± 2.0 °C using an ice bath. After 5 h of extraction, the supernatant was collected by centrifugation at 4800 rpm for 10 min. Ethanol was evaporated from the filtered extract, and the resulting sample was stored at −20 °C. The sample was dissolved in DMSO and diluted with phosphate-buffered saline (PBS) for the experiments.

### 2.3. Compositional Analysis of USHE

The composition of USHE was determined by following the below-mentioned methods. The total protein content in the sample was analyzed using Lowry’s method. Two different reagents (reagent A and reagent B) were pre-prepared in the method. Reagent A was prepared by dissolving 0.05 g of CuSO_4_·5H_2_O and 0.1 g of Na_3_C_6_H_5_O_7_·2H_2_O in 10 mL of distilled water (DW). Reagent B was prepared by dissolving 2 g of Na_2_CO_3_ and 0.4 g of NaOH in 100 mL of DW. Then Lowry’s reagent was prepared by mixing reagent A and reagent B in a 1:50 ratio. After that, 100 µL of 0, 0.2, 0.4, 0.6, 0.8, and 1 mg/mL BSA solutions and 100 µL of sample were mixed with 500 µL of Lowry’s reagent and incubated for 10 min at room temperature (RT). Then, 50 µL of 50% folin-ciocalteu reagent was mixed and incubated for another 10 min in RT. Lastly, the absorbance of standard series mixtures and the sample mixtures were checked at a 750 nm wavelength using SpectraMax M2 microplate reader (Molecular Devices, Silicon Valley, CA, USA). Total protein content was calculated in contrast to the standard curve. To measure the total protein content, 0.02, 0.04, 0.06, 0.08, and 0.10 mg/mL d-glucose series were prepared. Then 1 mL of each standard solution and 1 mL of 0.1 mg/mL sample solutions were mixed with 25 µL of 80% phenol solution and 2.5 mL of conc. H_2_SO_4_ in separate glass test tubes. After 30 min of incubation in the dark, the absorbance of each mixture was checked at a 480 nm wavelength using the microplate reader. Total carbohydrate content was calculated in contrast to the standard curve. The total phenol content in the sample was analyzed as below. In total, 100 µL of standard gallic acid (0, 0.02, 0.04, 0.08, and 0.1 mg/mL) and/or 0.1 mg/mL sample solutions were mixed with 100 µL of 95% ethanol and 500 µL of DW in e-tubes. Then 50 µL of 50% folin-ciocalteu reagent and 100 µL of 5% NaCO_3_ were mixed and incubated for 1 h in RT. Lastly, the absorbance of each mixture was checked at a 725 nm wavelength using the microplate reader. Total phenol content was calculated in contrast to the standard curve.

### 2.4. High-Performance Liquid Chromatography (HPLC) and Liquid Chromatography-Tandem Mass Spectrometry (LC-MS/MS) Analysis

HPLC analysis was conducted to determine the fucosterol content in the USHE. For this analysis, a Waters Alliance e2695 Separations Module (Milford, MA, USA) coupled with a Waters 2489 UV-vis Detector (USA) and a YMC Pack-Pro C18 column (4.6 × 250 mm, 5 μm) was used. The solvent system comprised 35% ethanol and 65% acetonitrile. Peaks observed in each sample at 210 nm and 35 °C were compared to a standard fucosterol sample dissolved in methanol. The most prominent phenolic compounds present in USHE were identified using a Shimadzu HPLC system, which included a CBM-20A system controller, a CTO-20AC column oven, an SPD-M20A photodiode detector, and an RID-10A refractive index detector. Separation was achieved with a Luna PFP (2) 100A column (150 × 3.0 mm, 3 μm particle size). Detection was performed at a wavelength of 270 nm, with a mixture of phenolic compounds used as the reference standard. LC-MS/MS analysis was implemented to identify the chemical components/prominent sulfoquinovosyl glycerolipids (SQGLs) in the USHE. The equipment used is a Waters Arc HPLC system (Waters Corp., USA) coupled with a Quattro Premier XE (Waters Corp., USA) mass spectrometer for this analysis.

### 2.5. Evaluation of Antioxidant Capacity

The DPPH radical scavenging activity of USHE was evaluated using the following method. In a 96-well plate, 100 µL of each USHE solution was mixed with 100 µL of a 150 µM DPPH solution. Distilled water (100 µL) was used as the control. The plate was then incubated at room temperature for 30 min. After incubation, the absorbance of each well was measured using the microplate reader. The DPPH scavenging activity was expressed as the percentage ratio of the absorbance difference between the control and sample to the absorbance of the control. In addition, the reducing capacity of the sample was directly measured by the ferric reducing antioxidant power (FRAP) assay, which is an essential indicator for assessing its effectiveness as a strong antioxidant [18]. The 10% ferric chloride solution was used to measure the FRAP of USHE. In brief, a mixture was prepared with 0.1 M phosphate buffer (pH 6.6–7.0), 1% potassium ferricyanide, and either the sample or distilled water (as a control) in a 3:5:2 ratio. This mixture was vortexed and incubated at 50 °C for 20 min. Following this, 10% trichloroacetic acid was added, and the mixture was centrifuged at 3000 rpm for 10 min. The supernatant was then mixed with distilled water and 10% ferric chloride in a 5:5:1 ratio, and the absorbance of the mixture was measured at 700 nm using a microplate reader. The FRAP of 125 µg/mL USHE in ascorbic acid equivalent was calculated by using a standard curve of the absorbance of different known concentrations of ascorbic acids.

### 2.6. Cell Culture

HaCaT keratinocytes were cultured in DMEM supplemented with 10% heat-inactivated FBS and a 1% penicillin/streptomycin antibiotic mixture. The cell culture was maintained in a humidified atmosphere with 5% CO_2_ at 37 °C. The cells were regularly sub-cultured until they reached exponential growth. During cell subculturing, cells were washed with PBS after the culture medium was removed. Following that, cells were allowed to detach with the aid of trypsin-EDTA (Gibco, Grand Island, NY, USA) for a brief period at 37 °C. Subsequently, the DMEM medium was used to neutralize the trypsin activity. The cells were gently moved, and the suspension was centrifuged. Next, the pellet was reconstituted in a new DMEM medium, and the concentration of cells was ascertained. The cells were seeded into new culture flasks for further growth. The harvested cells in the exponential growth phase, specifically between passages 3 and 10, were counted by using a hemocytometer (Paul Marienfeld GmbH & Co., KG, Lau-da-Königshofen, Germany) and seeded for the respective experiments.

### 2.7. Cell Viability Analysis

Cytotoxicity and the cytoprotective effect of the USHE on HaCaT keratinocytes against UVB stimulation were analyzed by following the MTT colorimetric cell viability analysis method [19]. The HaCaT keratinocytes were seeded at 1 × 10^5^ cells/well density in 96-well plates. The cell-seeded well plates were incubated for 24 h at the cell culture conditions mentioned in Section 2.5. Following the incubation, cells were treated with 15.6, 31.3, 62.5, 125, and 250 µg/mL concentrations of USHE to evaluate the cytotoxicity. Then, the cells were incubated for an additional 24 h under the same cell culture conditions. After that, 15 µL of 5 mg/mL of MTT reagent was added to each well and further incubated for 4 h in the dark. The cell culture media containing MTT was then carefully aspirated, and 100 µL of DMSO was added to each well. The mixture was gently shaken in the dark to dissolve the formazan crystals. After 30 min of shaking in the dark, absorbances were measured at 570 nm using the microplate reader. Then cells were stimulated with 40 mJ/cm^2^ of UVB exposure for 3.0 s at room temperature using an ultraviolet cross-linker (UVP CL-1000L, Upland, CA, USA) to analyze the protective effect of USHE on UVB-exposed HaCaT keratinocytes. Firstly, cultured cells were treated with 15.6, 31.3, 62.5, 125, and 250 µg/mL of USHE concentrations and incubated for an additional 2 h. Following that, cells were stimulated by UVB and incubated for 24 h. Then, 15 µL of 5 mg/mL MTT reagent was added to each well, and the remaining steps were carried out by following the same procedure described in the cytotoxicity analysis.

### 2.8. Analysis of Intracellular ROS Production

The intracellular ROS production in stimulated cells was analyzed by using a DCF-DA assay. In a nutshell, after 24 h of cell seeding, cells were treated with USHE concentrations of 15.6, 31.3, and 62.5 µg/mL and incubated for 2 h. Then, UVB stimulation was carried out to increase the intracellular ROS production in cells. After a 1 h stimulation period, DCF-DA was added to cells and fluorometric analysis was conducted by using the microplate reader with excitation and emission wavelengths of 485 and 528 nm, respectively. Furthermore, DCF-DA treated cells were evaluated by using a Thermo Fisher Scientific EVOS M5000 Imaging fluorescence microscope (Rockford, IL, USA) and a CytoFLEX flow cytometer (Beckman Coulter, Brea, CA, USA).

### 2.9. Nuclear Double Staining

Cells were seeded in 24-well plates and treated with selected concentrations of USHE for 2 h. After 24 h of UVB stimulation, cells were treated with an AO and EB 1:1 mixture (10 μg/mL). Stained cells were evaluated by imaging fluorescence microscope following 10 min of incubation.

### 2.10. Cell Cycle Analysis

Changes in the cell cycle were analyzed using the procedures outlined in a previous study [20]. Briefly, harvested cells were centrifuged at 1700 rpm for 5 min, and the resultant cell pellet was permeabilized in 70% ethanol at 4 °C overnight. Following a 5 min centrifugation at 1700 rpm, ethanol was removed by using suction. Then, cells were washed with PBS containing 2 mM of EDTA and centrifuged at 1500 rpm for 5 min. Subsequently, cells were resuspended in PBS containing EDTA, RNase, and propidium iodide and incubated at 37 °C for 30 min before being analyzed in the flow cytometer.

### 2.11. Annexin V and JC-1 Assays

The stimulated cells were harvested after a 6 h incubation period for the flow cytometry-based Annexin V assay to assess apoptosis by using an Annexin V-conjugated fluorescein isothiocyanate and propidium iodide kit (Invitrogen, CAT #V13242, Carlsbad, CA, USA). The cellular mitochondrial health status was evaluated using the MitoProbe^TM^ JC-1 assay kit (Invitrogen, Carlsbad, CA, USA) after harvesting stimulated cells following a 4 h incubation period. The manufacturer’s instructions were followed while conducting the analyses.

### 2.12. Western Blot Analysis

The harvested cells were lysed using NE-PER^®^ nuclear and cytoplasmic extraction kits and separately collected the cytoplasmic and nuclear proteins. Afterward, protein levels in the lysates were determined using the BCA protein assay kit, and 30 µg of protein from each lysate was electrophoresed. Tris-glycine SDS-PAGE was performed with 10% resolving gels and 5% stacking gels. The resolved proteins in the gels were then transferred into nitrocellulose membranes (Merck Millipore, Country Cork, Ireland) and blocked with 5% skim milk in TBST (Tris-buffered saline with Tween 20). Following this, the bands were sequentially incubated with primary (1:1000) and HRP-conjugated secondary (1:3000) antibodies. Lastly, bands were detected by using an enhanced chemiluminescence reagent (Cyanagen SRL, Bologna, Italy) on a Core Bio Davinch-ChemiTM imaging system (Seoul, Republic of Korea).

### 2.13. Statistical Analysis

The data are shown as mean ± standard error (SE), and the experiments were carried out in triplicate (n = 3). Significant differences among the data sets were evaluated using a one-way analysis of variance (ANOVA) followed by Duncan’s multiple range test, performed using IBM SPSS software (Version 24.0, Chicago, IL, USA). *p* ˂ 0.05 was considered statistically significant in this study.

## 3. Results

### 3.1. Extraction Yield and Proximate Composition of USHE and Identification of Active Compounds

The extraction yield of USHE was 29.50 ± 1.32%, and the proximate compositional analysis of USHE indicated a higher crude protein content (15.01 ± 1.06%) than carbohydrate content (9.83 ± 0.87%). The redox reactions in the folin-ciocalteu assay have been reported as a widely used method in studies to measure the total phenolic composition in foods or extracts [21]. Based on the present analysis, USHE contained 6.97 ± 0.36% of total phenolic compounds. HPLC analysis results revealed that the fucosterol content in USHE was 6.22 ± 0.06 mg/g (Figure S1). Furthermore, the main peaks in LC-MS/MS chromatography were identified as 1-O-Hexadecanoyl-3-O-(6′-sulfo-α-D-quinovopyranosyl) glycerol (Figure S3). The tandem mass analysis predicted other peaks to be related to isomer compounds. The high-resolution mass spectrum displayed *m*/*z* values of 527.2718, 553.3187, 555.3054, and 581.3199 ([M-H]), tentatively identified as 1-O-Tetradecanoyl-3-O-(6′-sulfo-α-D-quinovopyranosyl) glycerol, 1-O-(11-Hexadecenoyl)-3-O-(6′-sulfo-α-D-quinovopyranosyl) glycerol, 1-O-Hexadecanoyl-3-O-(6′-sulfo-α-D-quinovopyranosyl) glycerol, and 1-O-(9-Octadecenoyl)-3-O-(6′-sulfo-α-D-quinovopyranosyl) glycerol, respectively, based on high-resolution mass and AntiBase search (Table 1). Based on the previous study reported by Ashan J. and the team [13], we chose the most prominent phenolic mixture as a standard and performed HPLC analysis. As per the results, we identified Quinic acid, Salicylic acid, Protocatechuic acid, Caffeic acid, Vanillic acid, Ferulic acid, Syringic acid, Sinapic acid, and Syringin (Figure S2).

### 3.2. Antioxidant Capacity of USHE

**Figure 1:**
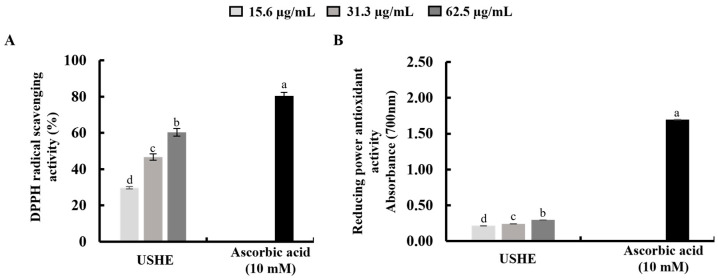
Antioxidant activities of USHE: (**A**) DPPH radical scavenging activity and (**B**) reducing power antioxidant activity. Values are expressed as the mean ± SE from triplicate experiments. Bars with different letters within the same graph represent significant differences (*p* < 0.05).

According to the findings, USHE indicated DPPH radical scavenging activity with a respective IC_50_ value of 49 ± 0.24 µg/mL (Figure 1A). The FRAP of the USHE was increased dose-dependently, with a maximum absorbance of 0.2927 at 62.5 μg/mL (Figure 1B). The reference standard (10 mM of ascorbic acid) was used to compare the sample activities [22]. As per the analysis, the FRAP of the 125 µg/mL of USHE is 71.29 µg/mL ascorbic acid equivalents.

### 3.3. Effect of USHE on Cell Viability and Intracellular ROS Production in UVB-Exposed HaCaT Keratinocytes

**Figure 2:**
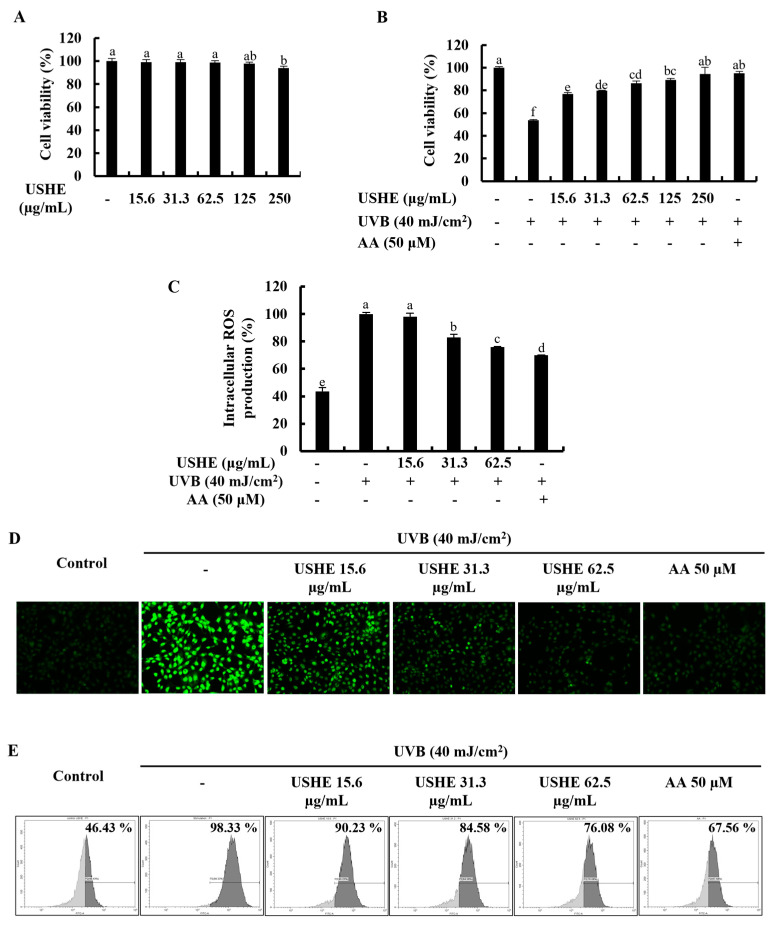
Analysis of the protective effects of USHE on UVB-exposed HaCaT keratinocytes. Evaluation of (**A**) cytotoxicity, (**B**) cell viability, and intracellular ROS production using (**C**) fluorometry, (**D**) fluorescence microscopy (Objective magnification of the instrument: 20×), and (**E**) flow cytometry. Values are expressed as the mean ± SE from triplicate experiments. Bars with different letters within the same graph represent significant differences (*p* < 0.05).

Significant cytotoxicity of USHE concentrations and the effect on cell viability of the UVB-exposed HaCaT keratinocytes were evaluated by using an MTT assay. As illustrated in Figure 2A, a significant cytotoxic effect on HaCaT keratinocytes was not observed for any of the tested USHE concentrations. UVB stimulation reduced the cell viability while markedly increasing viability with USHE in a dose-dependent manner (Figure 2B). However, 15.6, 31.3, and 62.5 µg/mL concentrations of USHE were selected to be used throughout the investigation due to the significant increase in cell viability. Figure 2C shows that intracellular ROS production in UVB-exposed HaCaT keratinocytes was dramatically raised, while it decreased with USHE. The ROS scavenging ability of USHE was further confirmed by DCF-DA fluorescence imaging and DCF-DA flow cytometric analysis. As depicted in Figure 2D, the intensity of green fluorescence elevated with UVB stimulation, while it dose-dependently reduced with USHE. The DCF-DA flow cytometry revealed that UVB radiation increased the number of cells with a higher fluorescence (*x*-axis), which is correlated with higher ROS production. The results were recorded as counts against FITC-A in the flow cytometric analysis (Figure 2E). However, the number of cells with higher fluorescence was diminished with USHE in a dose-dependent manner. A 50 µM concentration of AA was used as a positive control for the cell experiments.

### 3.4. Effect of USHE on Proportion of Apoptotic Cells in UVB-Exposed HaCaT Keratinocytes

**Figure 3:**
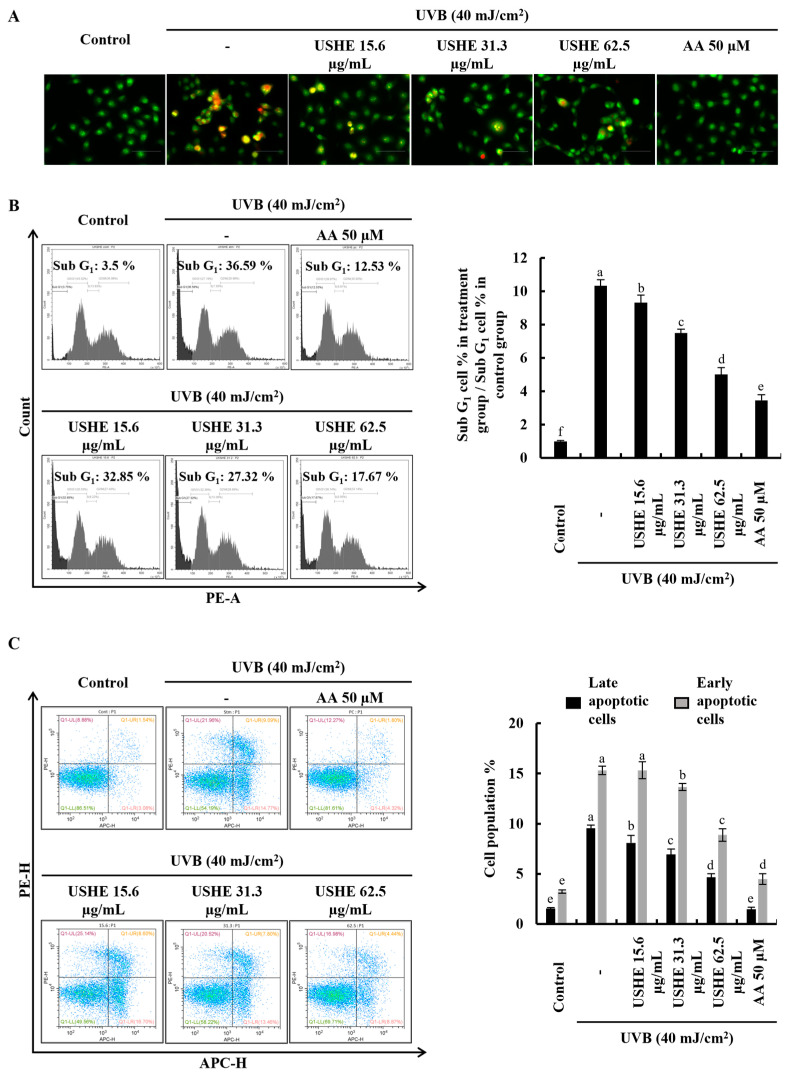
Effect of USHE on the apoptotic cell population in UVB-exposed HaCaT keratinocytes. (**A**) Nuclear morphology analysis using EB and AO double staining (Objective magnification of the instrument: 40×), (**B**) sub G_1_ apoptotic population analysis by flow cytometry using PI, and (**C**) early apoptosis investigation using flow cytometry Annexin V analysis. Values are expressed as the mean ± SE from triplicate experiments. Bars with different letters within the same graph represent significant differences (*p* < 0.05).

Apoptotic cell death is triggered by UVB-exposed oxidative stress in HaCaT keratinocytes [23]. Damaged membranes of late apoptotic and dead cells can be identified with EB staining. Figure 3A demonstrated an increase in the number of cells with orange-colored nuclei, which was subsequently decreased by USHE in a dose-dependent manner, indicating the attenuation of late apoptosis in UVB-exposed cells. In addition, cell cycle analysis was conducted to detect apoptosis and DNA damage after PI staining [20]. According to Figure 3B, the sub G_1_ apoptotic cell population (36.59 ± 1.1%) increased after UVB stimulation compared to the control group (3.5 ± 0.05%). However, the sub G_1_ apoptotic cell population was dose-dependently lowered with USHE. Annexin V flow cytometry analysis can be used to investigate the cellular early apoptosis. As shown in Figure 3C, the number of viable cells declined with UVB stimulation compared to the control group, increasing early apoptotic cells. Nevertheless, USHE notably reduced the early apoptotic cell population in a dose-dependent manner. The early apoptotic cell population was lowest at 62.5 µg/mL of USHE concentration.

### 3.5. Effect of USHE Against UVB-Exposed Oxidative Stress by Inhibiting Mitochondria-Mediated Apoptotic Signaling Pathway in HaCaT Keratinocytes

**Figure 4:**
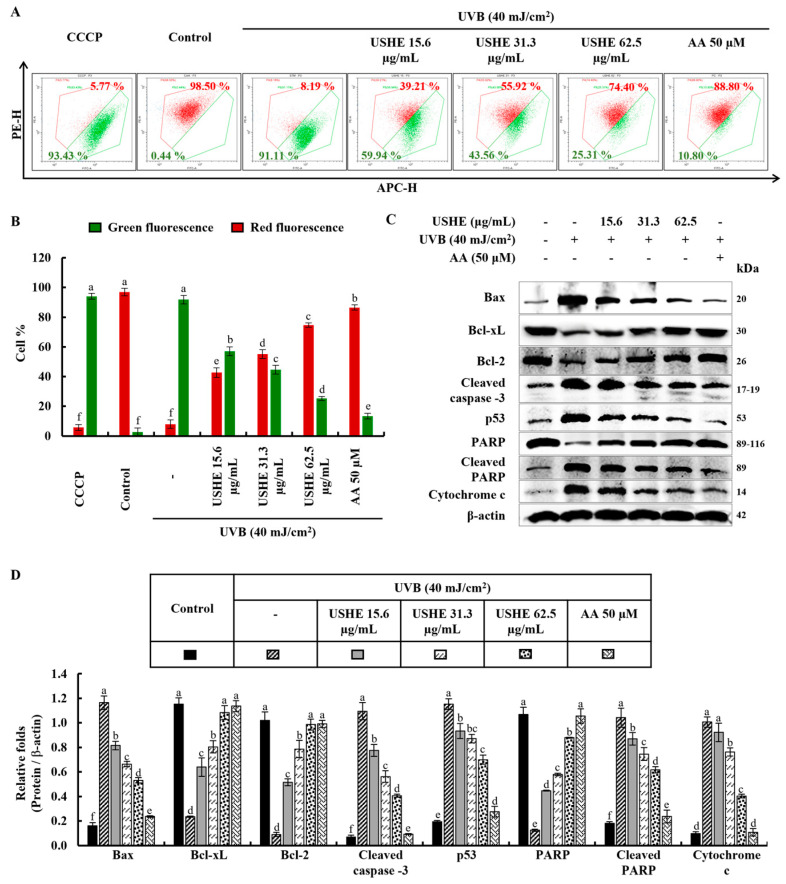
Effect of USHE on mitochondria-mediated apoptosis in UVB-exposed HaCaT keratinocytes. (**A**,**B**) Mitochondrial membrane potential analysis by JC-1 assays, with numbers indicating the percentage of cells. Western blot analysis of (**C**) mitochondria-mediated apoptotic pathway protein expression levels and (**D**) their relative fold changes. Values are expressed as the mean ± SE from triplicate experiments. Bars with different letters within the same graph represent significant differences (*p* < 0.05).

The JC-1 assay is used to evaluate the membrane potential and health of mitochondria in cells. Healthy, polarized mitochondria with stable membrane potential have a higher red/green ratio in the JC-1 assay. Reduced red/green ratios are indicative of mitochondrial depolarization, which may be a marker of early apoptosis or mitochondrial dysfunction. According to the findings of the study (Figure 4A,B), UVB-exposed cells exhibited a lower red/green ratio, indicating a significant presence of unhealthy mitochondria, similar to cells treated with the mitochondrial membrane potential disruptor, carbonyl cyanide m-chlorophenyl hydrazone (CCCP). However, USHE effectively increased the red/green ratio, suggesting the presence of healthy, polarized mitochondria with a stable membrane potential. Moreover, the release of chemicals from mitochondria, including cytochrome c, into the cytoplasm occurs when cells are stimulated by UVB due to an imbalance in the mitochondrial activities. This release leads to a series of reactions in the cell that subsequently cause apoptosis by activating several proteins and enzymes. The protein levels of Bcl-2-associated X (Bax), cleaved caspase-3, p53, cleaved PARP, and cytochrome c were upregulated by UVB exposure, while inhibiting anti-apoptotic mediators, including B-cell lymphoma (Bcl)-xL, Bcl-2, and PARP (Figure 4C). Interestingly, USHE improved the levels of Bcl-xL, Bcl-2, and PARP in a dose-dependent manner while downregulating levels of Bax, cleaved caspase-3, p53, cleaved PARP, and cytochrome c (Figure 4C). Relative folds of the above proteins are indicated in Figure 4D.

### 3.6. Effect of USHE on Nrf2/HO-1 Activation in UVB-Exposed HaCaT Keratinocytes

**Figure 5:**
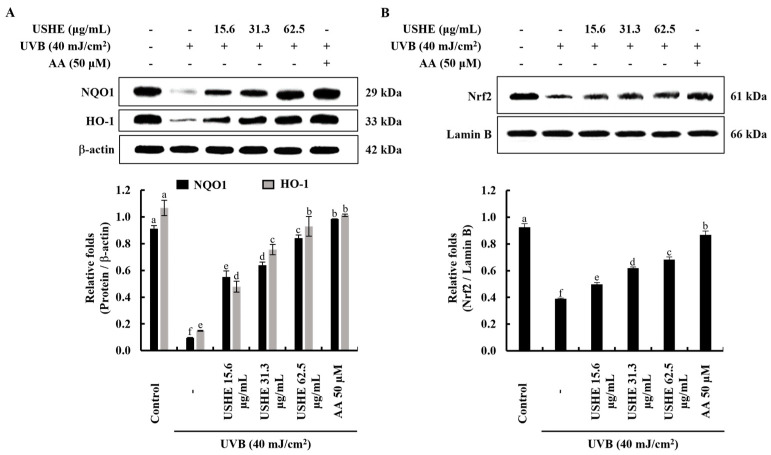
Effect of USHE on the activation of the Nrf2/HO-1 signaling pathway. Western blot analysis of (**A**) cytosolic HO-1, NQO1, and (**B**) nuclear translocated Nrf2 protein expression in UVB-exposed HaCaT keratinocytes. Values are expressed as the mean ± SE from triplicate experiments. Bars with different letters within the same graph represent significant differences (*p* < 0.05).

The Nrf2/HO-1 signaling pathway includes key factors that regulate the cellular oxidative stress response via inhibiting ROS generation. The findings from the Western blot analysis of protein expressions of cytosolic NQO1, HO-1, and nuclear translocated Nrf2 revealed the protective effect of USHE. According to Figure 5, the pretreatment with USHE increased the levels of cytosolic NQO1, HO-1, and nuclear translocated Nrf2 in UVB-exposed HaCaT keratinocytes in a dose-dependent manner.

## 4. Discussion

The present study demonstrates the photoprotective effect of USHE against UVB-induced oxidative stress and apoptosis in HaCaT keratinocytes. The significance of this research lies in its identification of USHE as a potential candidate for skin protection, particularly in the context of UVB exposure.

Conventionally various organic solvent extraction techniques have been employed to prepare extracts from marine resources [24,25]. However, recent studies have indicated the potential of ultrasonic-assisted extractions of seaweed to recover bioactive materials [13]. As we employed an ultrasonic-assisted ethanol extraction method in this study, we evaluated the total yield and the selected bioactive constituents in the same extracts reported previously. The results of the current study indicated that USHE has a 29.50 ± 1.32% yield (*w*/*w*), confirming ultrasonic-assisted ethanol extraction method has the potential to obtain higher yields compared to the ethanol extracts [14]. Various active components, including phenolic compounds, flavonoids and their derivatives, lignans, terpenes, tannins, sulfolipids and phospholipids, carboxylic acids, fatty acids, sugars, few other organic compounds in ultrasonic extract of *S. horneri*, have been identified in a previous study [13]. Interestingly, we have identified the most prominent phenolic compounds, namely, Quinic acid, Salicylic acid, Protocatechuic acid, Caffeic acid, Vanillic acid, Ferulic acid, Syringic acid, Sinapic acid, and Syringin in the present study. The total phenolic compounds were 6.97 ± 0.36% in USHE on a dry basis (%). Additionally, four SQGLs in the USHE sample were identified by using MS analysis compared with previously reported data by Jun and Z. Cui et al. [26,27]. The unique structure of SQGLs allows them to integrate into cellular membranes, contributing to membrane stability and protecting against lipid peroxidation, a common result of oxidative stress [28]. These compounds have potential applications in cosmeceuticals, where they can combat skin aging and enhance skin integrity due to their antioxidant effects. One of our previous reports indicated that fucosterol from 70% ethanol extract of *S. horneri* exhibited potential antioxidant and anti-inflammatory effects against TNF-α/IFN-γ-stimulated HDF cells by regulating the Nrf2/HO-1, NF-κB, and MAPK signaling pathways [14,29]. Moreover, we observed a promising allergic potential of fucosterol derived from the same extract in in vitro as well as in vivo studies [16]. In addition, the intracellular ROS scavenging effect of fucosterol isolated from *S. crassifolium* has been reported [30]. As one of our focuses in this method is to maximize the yield of fucosterol while reducing the extraction time, we conducted a separate HPLC analysis to identify and quantify fucosterol in the USHE sample. Interestingly, the analysis results indicated the fucosterol content in USHE was 6.22 ± 0.06 mg/g.

One of the previous studies indicated the strong DPPH and ABTS radical scavenging activity and the presence of fucosterol in *S. horneri* extract [14]. Moreover, many other studies have confirmed the antioxidative potential of *S. horneri* extracts [15,31]. The antioxidant capacity of the USHE was determined by DPPH radical scavenging activity and FRAP assay. The findings of the study showed that USHE indicated DPPH radical scavenging activity with a respective IC_50_ value of 49 ± 0.24 µg/mL. In addition, the FRAP assay is used to measure antioxidant power based on the reduction of ferric to ferrous ions at a low pH [32]. According to the FRAP assay, there was a significant and dose-dependent increase with a maximum absorbance of 0.2927 at 62.5 μg/mL. The FRAP of the 125 µg/mL of USHE is 71.29 µg/mL ascorbic acid equivalents. Based on the promising radical scavenging capacity and antioxidant power of USHE, further analysis was carried out to assess the protective effect of USHE against oxidative stress in UVB-exposed HaCaT keratinocytes.

In this study, different concentrations of USHE were used to examine the effect on the cell viability of UVB-exposed HaCaT keratinocytes. The findings of the study revealed that there is a significant increment in cell viability with USHE dose-dependently. The observed protective effects of USHE against UVB-induced oxidative stress in HaCaT keratinocytes were further corroborated by investigations of intracellular ROS levels. The findings revealed that UVB exposure resulted in a marked increase in ROS production, which aligns with previous studies that report ROS as a significant contributor to damage in keratinocytes under UV radiation [33]. Importantly, USHE treatment effectively reduced intracellular ROS levels in UVB-exposed cells, validating its efficacy as an antioxidant agent. The analysis also included a nuclear double-staining experiment using EB and AO, showcasing a dose-dependent reduction in both early and late apoptotic cell populations after treatment with USHE. The same changes in nuclear double staining in human skin cells under UVB and sunlight exposure have been reported [34]. Upon cell cycle investigation of the present study, UVB stimulation increased the cell accumulation in the Sub-G_1_ phase, while it significantly decreased it by USHE doses in contrast to the stimulation group of HaCaT keratinocytes. The findings strongly supported the recovery of physiological differentiation of the UVB-exposed HaCaT keratinocytes by the USHE.

Mitochondrial alterations and their role in apoptosis have been known for several years. In apoptotic experimental models, a loss of mitochondrial membrane potential (ΔΨm) is a hallmark of apoptosis [35]. As far as ΔΨm plays an important role, the JC-1 analysis technique is frequently used to assess cells with high or low ΔΨm. Previous research has explored that UVB-exposed HaCaT keratinocytes show a large proportion of unhealthy mitochondria, similar to the CCCP group [36]. Results obtained from JC-1 analysis in the present study allowed us to identify two identical populations with different ΔΨm, a first population with high ΔΨm and another one with low ΔΨm. USHE gradually increases the ΔΨm in contrast to the CCCP group in a dose-dependent manner.

Our investigation involved examining the role of the tumor suppressor protein p53, which is crucial in mediating UVB-induced apoptosis in these cellular contexts [37]. Mitochondrial fission is fundamental for apoptosis, which facilitates the release of pro-apoptotic factors, such as cytochrome c, from the mitochondrial intermembrane space [38]. This release triggers a cascade of events leading to cell death. The increase of cytochrome c, Bax, cleaved caspase-3, cleaved PARP, and p53 levels in UVB-exposed HaCaT keratinocytes, with a decrease of Bcl-2 and Bcl-xL levels, suggests the initiation of apoptosis in this study. Moreover, PARP cleavage took place with the UVB stimulation. Similarly, apoptogenic protein expressions have been shown due to UVB stimulation in one of the prior studies [39]. The results of the present study showed a dose-dependent reduction in apoptotic molecular mediators after treatment with USHE, demonstrating its protective properties against UVB-exposed oxidative stress in HaCaT keratinocytes. Through flow cytometric and Western blot analyses, we observed significant alterations in apoptotic signaling pathways with USHE sample treatments in UVB-exposed oxidative stress in HaCaT keratinocytes.

The activation of Nrf2 to promote the expression of HO-1 and NQO1 is considered a promising therapeutic strategy to mitigate harmful cellular responses [40]. Nrf2 remains bound to the cytoplasmic inhibitor Keap1. Upon stimulation, Nrf2 undergoes translocation to the nucleus, initiating the transcription of various antioxidant genes, such as HO-1 and NQO1. Induction of HO-1 and NQO1 in UVB-exposed cells suppresses increased intracellular ROS levels, indicating an antioxidative impact [41]. The findings indicate that USHE enhances Nrf2 activation and its nuclear translocation. This is supported by the increased levels of HO-1 and NQO1, which further highlight the antioxidant potential of USHE. Collectively, these results show that USHE boosts Nrf2 activation and regulates the expression of antioxidant enzymes such as HO-1 and NQO1 in UVB-exposed HaCaT keratinocytes. Given its antioxidant properties and the presence of beneficial phytochemicals, USHE holds promise as a valuable ingredient for skincare products and cosmeceuticals. Future studies should explore its broader bioactivity through both in vitro and in vivo evaluations for various diseases.

## 5. Conclusions

Based on the present study, USHE effectively inhibits oxidative stress by reducing ROS production in UVB-exposed HaCaT keratinocytes. Furthermore, USHE indicates a protective effect by downregulating mitochondria-mediated apoptotic signaling and nuclear damage. Therefore, USHE possesses antioxidant potential against photodamage of the skin and can be utilized for the development of nutraceuticals/cosmeceuticals for UVB protection. Furthermore, this study suggests that USHE could be further investigated through in vitro and in vivo studies to evaluate its application as an ingredient in cosmeceuticals with antioxidant capabilities.

## Figures and Tables

**Table 1:** Metabolite profiling of USHE.

RT (min)	*m*/*z* ([M-H])	Formula	Δppm	Compound
16.12	527.2518	C_23_H_43_O_11_S	−0.0192	1-O-Tetradecanoyl-3-O-(6′-sulfo-a-D-quinovopyranosyl) glycerol
17.17	553.3187	C_25_H_45_O_11_S	−0.0504	1-O-(11-Hexadecenoyl)-3-O-(6′-sulfo-a-D-quinovopyranosyl) glycerol
19.02	555.3054	C_25_H_47_O_11_S	−0.0215	1-O-Hexadecanoyl-3-O-(6′-sulfo-a-D-quinovopyranosyl) glycerol
20.13	581.3199	C_27_H_49_O_11_S	−0.0007	1-O-(9-Octadecenoyl)-3-O-(6′-sulfo-a-D-quinovopyranosyl) glycerol

## Data Availability

Data is contained within the article and Supplementary Materials.

## Supplementary Materials

The following supporting information can be downloaded at https://www.mdpi.com/article/10.3390/antiox13111342/s1, Figure S1: HPLC-chromatograms of (A) standard fucosterol and (B) USHE, Figure S2: HPLC-chromatogram of (A) flavonoid reference standard and (B) SBH, Figure S3: The LC-MS/MS result of extract of *S. horneri*.

## References

[1] Taylor S.C., Alexis A.F., Armstrong A.W., Fuxench Z.C.C., Lim H.W. (2022). Misconceptions of photoprotection in skin of color. J. Am. Acad. Dermatol..

[2] Lucas R., Yazar S., Young A., Norval M., De Gruijl F., Takizawa Y., Rhodes L., Sinclair C., Neale R. (2019). Human health in relation to exposure to solar ultraviolet radiation under changing stratospheric ozone and climate. Photochem. Photobiol. Sci..

[3] Mapoung S., Arjsri P., Thippraphan P., Semmarath W., Yodkeeree S., Chiewchanvit S., Piyamongkol W., Limtrakul P. (2020). Photochemoprotective effects of Spirulina platensis extract against UVB irradiated human skin fibroblasts. S. Afr. J. Bot..

[4] Leal A.C., Corrêa M.P., Holick M.F., Melo E.V., Lazaretti-Castro M. (2021). Sun-induced production of vitamin D3 throughout 1 year in tropical and subtropical regions: Relationship with latitude, cloudiness, UV-B exposure and solar zenith angle. Photochem. Photobiol. Sci..

[5] Xiao Z., Yang S., Liu Y., Zhou C., Hong P., Sun S., Qian Z.-J. (2022). A novel glyceroglycolipid from brown algae Ishige okamurae improve photoaging and counteract inflammation in UVB-induced HaCaT cells. Chem.-Biol. Interact..

[6] Lennicke C., Cochemé H.M. (2021). Redox metabolism: ROS as specific molecular regulators of cell signaling and function. Mol. Cell.

[7] Krylatov A.V., Maslov L.N., Voronkov N.S., Boshchenko A.A., Popov S.V., Gomez L., Wang H., Jaggi A.S., Downey J.M. (2018). Reactive oxygen species as intracellular signaling molecules in the cardiovascular system. Curr. Cardiol. Rev..

[8] Hong Y., Boiti A., Vallone D., Foulkes N.S. (2024). Reactive oxygen species signaling and oxidative stress: Transcriptional regulation and evolution. Antioxidants.

[9] Sinenko S.A., Starkova T.Y., Kuzmin A.A., Tomilin A.N. (2021). Physiological signaling functions of reactive oxygen species in stem cells: From flies to man. Front. Cell Dev. Biol..

[10] Pisoschi A.M., Pop A. (2015). The role of antioxidants in the chemistry of oxidative stress: A review. Eur. J. Med. Chem..

[11] Cavinato M., Waltenberger B., Baraldo G., Grade C.V., Stuppner H., Jansen-Dürr P. (2017). Plant extracts and natural compounds used against UVB-induced photoaging. Biogerontology.

[12] Karthikeyan A., Joseph A., Nair B.G. (2022). Promising bioactive compounds from the marine environment and their potential effects on various diseases. J. Genet. Eng. Biotechnol..

[13] Javed A., Alam M.B., Naznin M., Ahmad R., Lee C.H., Kim S., Lee S.-H. (2024). RSM-and ANN-Based Multifrequency Ultrasonic Extraction of Polyphenol-Rich Sargassum horneri Extracts Exerting Antioxidative Activity via the Regulation of MAPK/Nrf2/HO-1 Machinery. Antioxidants.

[14] Kirindage K.G.I.S., Jayasinghe A.M.K., Han E.-J., Jee Y., Kim H.-J., Do S.G., Fernando I.P.S., Ahn G. (2022). Fucosterol isolated from dietary brown alga Sargassum horneri protects TNF-α/IFN-γ-stimulated human dermal fibroblasts via regulating Nrf2/HO-1 and NF-κB/MAPK pathways. Antioxidants.

[15] Lee J.H., Kim H.J., Jee Y., Jeon Y.-J., Kim H.J. (2020). Antioxidant potential of Sargassum horneri extract against urban particulate matter-induced oxidation. Food Sci. Biotechnol..

[16] Jayasinghe A.M.K., Yang H.-W., Kirindage K.G.I.S., Jung K., Je J.-G., Wang L., Kim K.-N., Ahn G. (2024). Fucosterol isolated from Sargassum horneri attenuates allergic responses in immunoglobulin E/bovine serum albumin-stimulated mast cells and passive cutaneous anaphylaxis in mice. Int. Immunopharmacol..

[17] Kirindage K.G.I.S., Jayasinghe A.M.K., Ko C.-I., Ahn Y.-S., Heo S.-J., Oh J.-Y., Kim E.-A., Cha S.-H., Ahn G. (2024). Unveiling the Potential of Ultrasonic-Assisted Ethanol Extract from Sargassum horneri in Inhibiting Tyrosinase Activity and Melanin Production in B16F10 Murine Melanocytes. Front. Biosci.-Landmark.

[18] Firuzi O., Lacanna A., Petrucci R., Marrosu G., Saso L. (2005). Evaluation of the antioxidant activity of flavonoids by “ferric reducing antioxidant power” assay and cyclic voltammetry. Biochim. Biophys. Acta (BBA)-Gen. Subj..

[19] Kirindage K.G.I.S., Jayasinghe A.M.K., Han E.-J., Han H.-J., Kim K.-N., Wang L., Heo S.-J., Jung K.-S., Ahn G. (2023). Phlorofucofuroeckol-A refined by edible brown algae Ecklonia cava indicates anti-inflammatory effects on TNF-α/IFN-γ-stimulated HaCaT keratinocytes and 12-O-tetradecanoylphorbol 13-acetate-induced ear edema in BALB/c mice. J. Funct. Foods.

[20] Kirindage K.G.I.S., Jayasinghe A.M.K., Cho N., Cho S.H., Yoo H.M., Fernando I.P.S., Ahn G. (2022). Fine-dust-induced skin inflammation: Low-molecular-weight fucoidan protects keratinocytes and underlying fibroblasts in an integrated culture model. Mar. Drugs.

[21] Pérez M., Dominguez-López I., Lamuela-Raventós R.M. (2023). The chemistry behind the folin–ciocalteu method for the estimation of (poly) phenol content in food: Total phenolic intake in a mediterranean dietary pattern. J. Agric. Food Chem..

[22] Fernando I.P.S., Kirindage K.G.I.S., Jayasinghe A.M.K., Han E.J., Dias M.K.H.M., Kang K.P., Moon S.I., Shin T.S., Ma A., Jung K. (2022). Hot Water Extract of Sasa borealis (Hack.) Makino & Shibata Abate Hydrogen Peroxide-Induced Oxidative Stress and Apoptosis in Kidney Epithelial Cells. Antioxidants.

[23] Long Y., Wang W., Zhang Y., Du F., Zhang S., Li Z., Deng J., Li J. (2023). Photoprotective effects of dendrobium nobile lindl. polysaccharides against UVB-Induced oxidative stress and apoptosis in HaCaT Cells. Int. J. Mol. Sci..

[24] Abhari K., Khaneghah A.M. (2020). Alternative extraction techniques to obtain, isolate and purify proteins and bioactive from aquaculture and by-products. Advances in Food and Nutrition Research.

[25] Airanthi M.W.A., Hosokawa M., Miyashita K. (2011). Comparative antioxidant activity of edible Japanese brown seaweeds. J. Food Sci..

[26] Cho J.H., Kim D.H., Lee J.S., Seo M.-S., Kim M.E., Lee J.S. (2022). *Sargassum horneri (Turner)* C. Agardh extract regulates neuroinflammation in vitro and in vivo. Curr. Issues Mol. Biol..

[27] Cui Z., Li Y.S., Liu H.B., Yuan D., Lu B.R. (2001). Sulfoglycolipid from the marine brown alga Sargassum hemiphylum. J. Asian Nat. Prod. Res..

[28] Plouguerné E., da Gama B.A., Pereira R.C., Barreto-Bergter E. (2014). Glycolipids from seaweeds and their potential biotechnological applications. Front. Cell. Infect. Microbiol..

[29] Kim M.J., Jo H.G., Ramakrishna C., Lee S.-J., Lee D.-S., Cheong S.H. (2021). Anti-inflammatory and antioxidant activities of Sargassum horneri extract in RAW264. 7 macrophages. Phys. Act. Nutr..

[30] Chi H.K., Van N.T.H., Hang T.T.N., Duc T.M., Ha T.T.H. (2020). Intracellular reactive oxygen species scavenging effect of fucosterol isolated from the brown alga Sargassum crassifolium in Vietnam. Vietnam J. Mar. Sci. Technol..

[31] Park P.J., Shahidi F., Jeon Y.J. (2004). Antioxidant activities of enzymatic extracts from an edible seaweed Sargassum horneri using ESR spectrometry. J. Food Lipids.

[32] Benzie I.F., Strain J.J. (1996). The ferric reducing ability of plasma (FRAP) as a measure of “antioxidant power”: The FRAP assay. Anal. Biochem..

[33] Jin G.-H., Liu Y., Jin S.-Z., Liu X.-D., Liu S.-Z. (2007). UVB induced oxidative stress in human keratinocytes and protective effect of antioxidant agents. Radiat. Environ. Biophys..

[34] Dubey D., Chopra D., Singh J., Srivastav A.K., Kumari S., Verma A., Ray R.S. (2017). Photosensitized methyl paraben induces apoptosis via caspase dependent pathway under ambient UVB exposure in human skin cells. Food Chem. Toxicol..

[35] Lugli E., Troiano L., Ferraresi R., Roat E., Prada N., Nasi M., Pinti M., Cooper E.L., Cossarizza A. (2005). Characterization of cells with different mitochondrial membrane potential during apoptosis. Cytom. Part A.

[36] Martins W.K., Costa É.T., Cruz M.C., Stolf B.S., Miotto R., Cordeiro R.M., Baptista M.S. (2015). Parallel damage in mitochondrial and lysosomal compartments promotes efficient cell death with autophagy: The case of the pentacyclic triterpenoids. Sci. Rep..

[37] Ziegler A., Jonason A.S., Leffellt D.J., Simon J.A., Sharma H.W., Kimmelman J., Remington L., Jacks T., Brash D.E. (1994). Sunburn and p53 in the onset of skin cancer. Nature.

[38] Landes T., Martinou J.-C. (2011). Mitochondrial outer membrane permeabilization during apoptosis: The role of mitochondrial fission. Biochim. Biophys. Acta (BBA)-Mol. Cell Res..

[39] Salucci S., Burattini S., Curzi D., Buontempo F., Martelli A.M., Zappia G., Falcieri E., Battistelli M. (2014). Antioxidants in the prevention of UVB-induced keratynocyte apoptosis. J. Photochem. Photobiol. B Biol..

[40] Dinkova-Kostova A.T., Copple I.M. (2023). Advances and challenges in therapeutic targeting of NRF2. Trends Pharmacol. Sci..

[41] Zhang L., Liang S., Zhang Z., Wang K., Cao J., Yao M., Qin L., Qu C., Miao J. (2023). Protective Effects of ζ-Carotene-like Compounds against Acute UVB-Induced Skin Damage. Int. J. Mol. Sci..

