# Supplementary material for: Photoprotective Effect of Ultrasonic-Assisted Ethanol Extract from Sargassum horneri on UVB-Exposed HaCaT Keratinocytes
Source: Antioxidants (Basel). 2024 Nov 1;13(11):1342. doi: 10.3390/antiox13111342 (PMC11591288; doi:10.3390/antiox13111342)
Supplement: Supplementary file 1 [file antioxidants-13-01342-s001.zip › antioxidants-3269001-supplementary.pdf]

*Supplementary document*

# Photoprotective Effect of Ultrasonic-assisted Ethanol Extract from *Sargassum horneri* on UVB-exposed HaCaT keratinocytes

***High-performance liquid chromatographic (HPLC) analysis***

HPLC analysis was conducted to identify the amount of fucosterol, where the samples contained a considerable number of phenolic compounds and to identify selected phenolic compound in USHE.

***Equipment and conditions in the evaluation of fucosterol***

Instrument : Waters Alliance e2695 Separations Module, USA. Waters 2489 UV/Vis Detector, USA

Column : YMC Pack-Pro C18 column (4.6 × 250 mm, 5 μm)

Operational temperature : 35 ℃

Injection (loop) volume : 10 μL

Mobile phase : Isocritic conditions [A: methanol, B: acetonitrile]

Flow rate : 1.1 mL/min

Detector wavelength : UV 210 nm

***Calibration curve of standard***

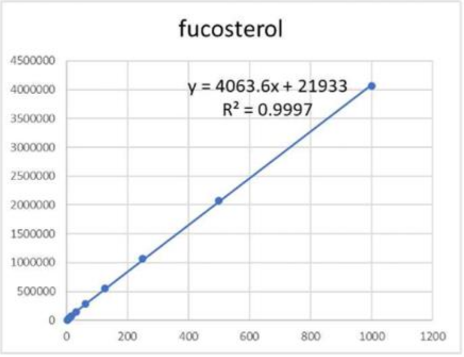


**A**

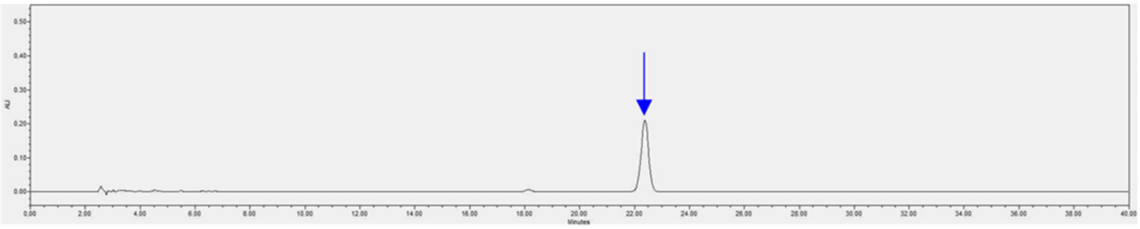

**B**

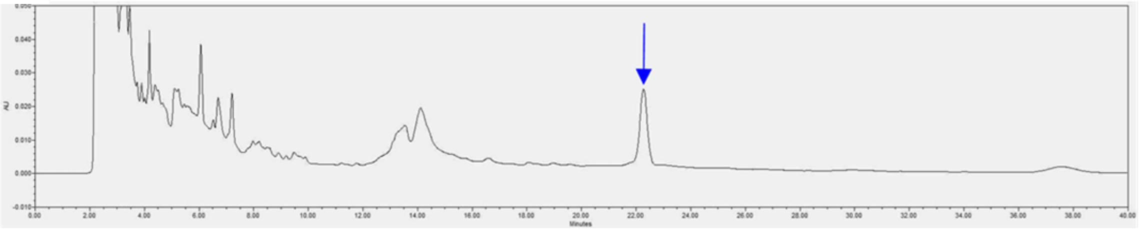

**Figure S1.** HPLC- chromatograms of (A) standard fucosterol and, (B) USHE.

*Equipment and conditions in the evaluation of selected phenolic compounds*

Instrument: Shimadzu HPLC consisting of a CBM-20A system controller, CTO-20AC column oven, SPD-M20A photodiode array detector, and a RID-10A refractive index detector

Column: Luna PFP(2) 100A (150 × 3.0 mm, 3 μm)

Injection (loop) volume : 10 μL

Mobile phase:

A: 0.1% formic acid in methanol

B: 0.1% formic acid in water

Gradient:

80% A (20% B) decreased to 35% A from 0 to 110 min; decreased to 0% A from 110 to 112 min, and kept at 0% A for 38 min; increased to 100% A from 130 to 132 min, and kept at 100% A for 16 min.

Flow rate: 0.34 mL/min

Detector wavelength: 270 nm

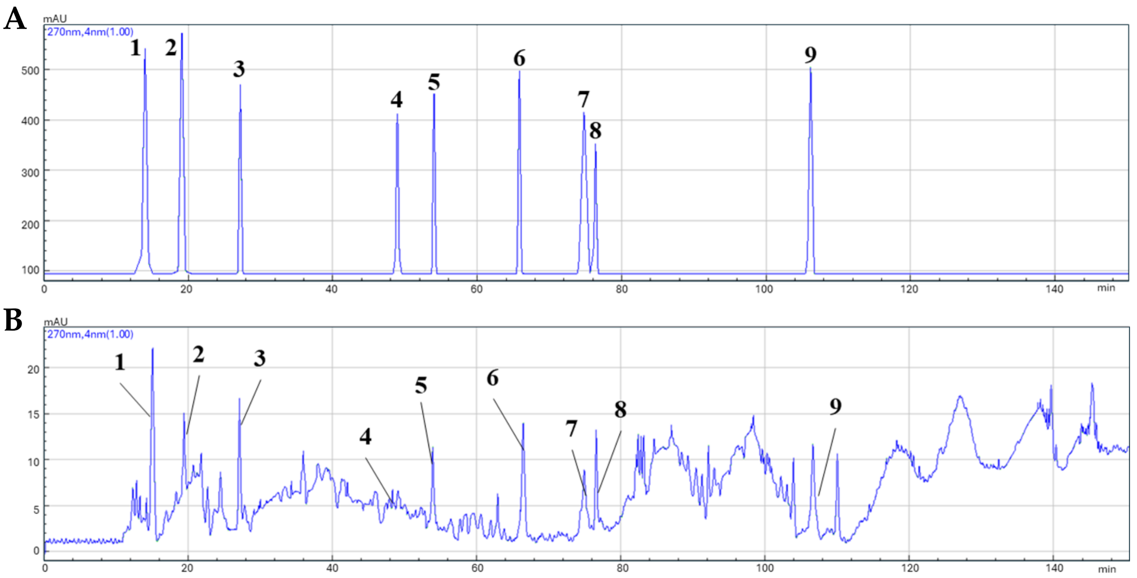


**Figure S2.** HPLC- chromatogram of (A) flavonoid reference standard and (B) SBH. [1. Quinic acid (standard: sigma 1594506), 2. Salicylic acid (standard: sigma 247588), 3. Protocatechuic acid (standard: sigma 03930590), 4. Caffeic acid (standard: sigma C0625), 5. Vanillic acid (standard: sigma H36001), 6. Ferulic acid (standard: sigma 128708), 7. Syringic acid (standard: sigma S6881), 8. Sinapic acid (standard: sigma 530-59-6), 9. Syringin (standard: sigma 90974).]

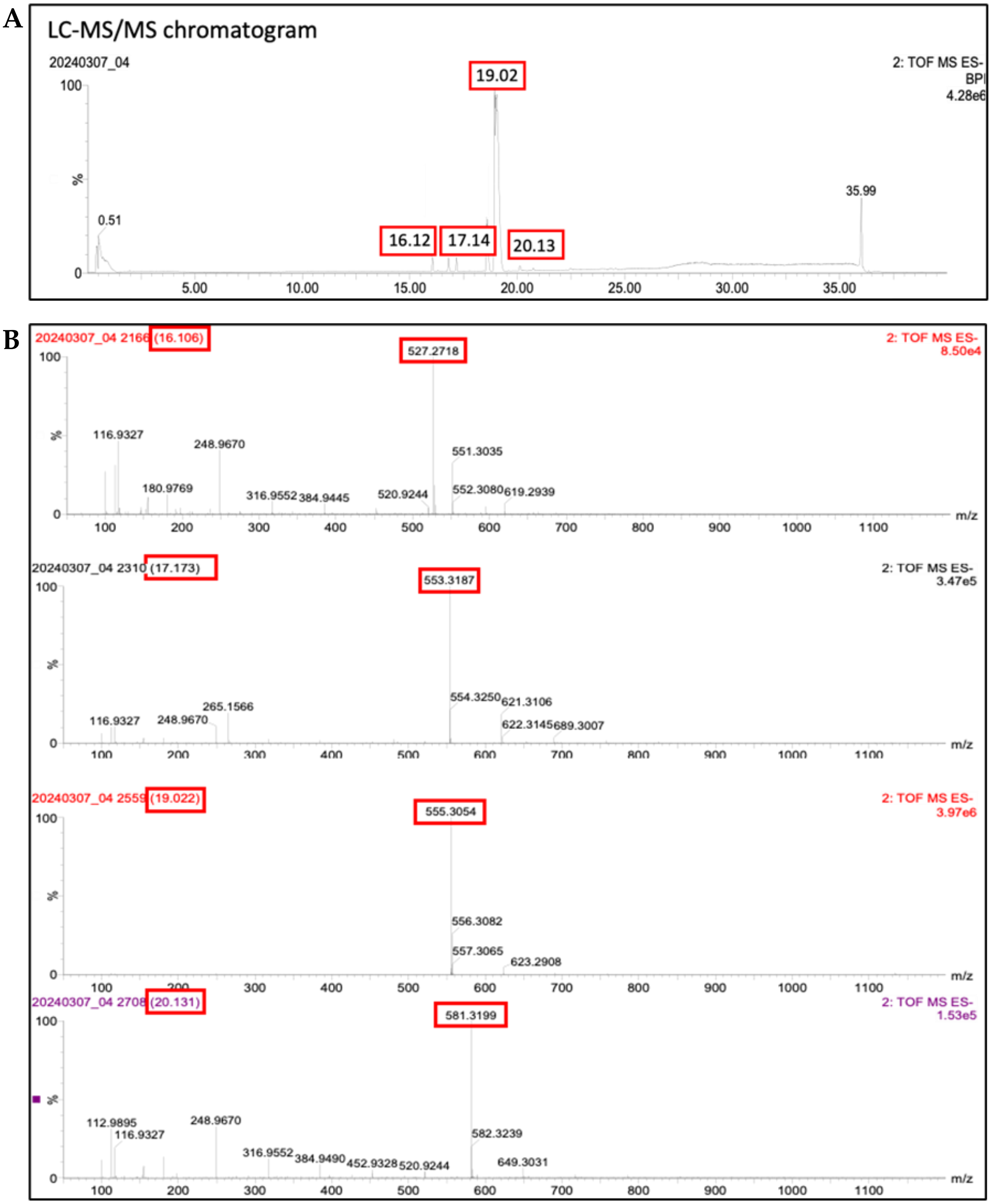


**Figure S3.** The LC-MS/MS result of extract of S. Horneri. (A) Mass chromatogram of the extract at negative ion mode. (B) High-resolution mass spectrum of 4 sulfoglycolipid compounds.
